# Endoscopic management of maxillary sinus inverted papilloma attachment sites to minimize disease recurrence

**DOI:** 10.1186/s40463-018-0271-1

**Published:** 2018-04-04

**Authors:** Vincent Wu, Jennifer Siu, Jonathan Yip, John M. Lee

**Affiliations:** 1 School of Medicine, Faculty of Health Sciences, Queen’s University, Kingston, Ontario Canada; 2 Division of Rhinology, Department of Otolaryngology – Head and Neck Surgery, St. Michael’s Hospital, University of Toronto, Toronto, Ontario Canada; 3 Li Ka Shing Knowledge Institute, St. Michael’s Hospital, Toronto, Ontario Canada

**Keywords:** Endoscopic, Sinus surgery, Maxillary sinus, Inverted papilloma, Prognosis, Recurrence, Attachment site, Multifocal, Medial maxillectomy

## Abstract

**Background:**

Inverted papillomas (IPs) are benign neoplasms, most commonly arising from the mucosal lining of the maxillary sinus. IPs can have single or multifocal sites of attachment. Although pedicle location is an important factor to consider in surgical planning, it is less clear whether the location or number of IP attachment sites hold any prognostic value. Herein, we aimed to determine the prognostic significance of the number and location of attachment sites of IPs originating from the maxillary sinus when managed by a pure endoscopic approach.

**Methods:**

This was a single-center, single-surgeon retrospective chart review. Patients with maxillary sinus IPs who were managed by endoscopic approaches only, from January 1, 2010 to June 30, 2016, were identified. Demographic data, operative technique, number and location of IP attachment sites, follow-up duration, recurrence, and presence of malignant transformation were captured.

**Results:**

Twenty-eight maxillary IP patients (61% males) were included, with a mean age of 54.9 (standard deviation (SD): 16.5) years. Approximately 36% of patients were referred from other institutions for management of recurrent IPs after failing previous surgical treatment. All patients were managed with an endoscopic approach, and all required an endoscopic medial maxillectomy to facilitate access to the maxillary sinus. At a mean follow-up of 31.1 (SD: 22.6) months, there were no recurrences identified. IPs with single (46%) and multifocal (54%) attachments were predominately to the medial and lateral walls. Maxillary IPs with multifocal attachments most frequently involved 2-3 walls of the sinus. Osteitis (36%) was commonly seen.

**Conclusion:**

IPs originating from the maxillary sinus frequently had multifocal attachments, but this did not impact disease recurrence. Despite the surgical challenges of accessing all of the maxillary sinus walls, IPs originating from the maxillary sinus can be effectively managed via a pure endoscopic approach.

## Background

Inverted papillomas (IPs) are benign neoplasms arising from the mucosal lining of the nasal cavity and paranasal sinuses [1]. IPs can have either single or multifocal sites of origin, with a recurrence rate ranging from 14 to 25% if surgical resection is not complete [2, 3]. This is of clinical significance as IPs are associated with a 5 – 15% malignant transformation rate to squamous cell carcinoma [4, 5].

The majority of IPs develop within the maxillary sinus, often originating from the medial wall [6–8]. Invasion into adjacent structures is the main complication of disease progression and can involve the orbit, lacrimal system, and skull base [9–12]. These tumors also have a tendency to erode and re-model bone, leading to devastating sequelae [13, 14].

Surgery is the mainstay of treatment for IPs and historically have included several approaches: 1) non-endoscopic endonasal, 2) limited external (i.e. Caldwell-Luc), 3) radical external (i.e. lateral rhinotomy or midfacial degloving with en bloc resection), and 4) endoscopic endonasal [5, 7, 12, 15]. A pedicle-oriented strategy is currently widely implemented in the resection of IPs with the surgical approach employed specific to the pedicle location. It has been reported that the endoscopic approach alone was insufficient in reaching all maxillary IPs pedicle sites, especially with IPs that originated from the lateral, anterior, and inferior sinus walls [7]. In these instances, external approaches such as the Caldwell-Luc were required for complete resection [7]. However, with advancements in endoscopic technologies and techniques, tumors originating from the maxillary sinus have increasingly been managed by a pure endoscopic approach alone.

Although the pedicle location is an important factor to consider in surgical planning, it is less clear whether the location or number of IP attachment sites hold any prognostic value. Herein, we aimed to determine the prognostic significance of the number and location of attachment sites of IPs originating from the maxillary sinus, when managed by a pure endoscopic approach. We hypothesize that IPs of multiple attachment sites are associated with increased recurrence.

## Methods

### Patient selection

We carried out a single surgeon (JML), single centre retrospective chart review. All patients who had resection for IPs originating only from the maxillary sinus, from January 1, 2010 to June 30, 2016, were included in the study. Patients who had expressed prior wishes not to be included in any clinical research at our academic centre were excluded. There were no other exclusion criteria.

Patient charts including pre-operative consults, operative notes, and post-operative follow-up reports were obtained and reviewed. All operative notes were dictated by the staff surgeon (JML). Demographic data, history of previous IP resection, surgical technique(s), presence of any perioperative complication(s), length of stay (LOS), and follow-up length were extracted.

### Surgical approach

Once pathology of IP was confirmed via intraoperative frozen sections, the mass was resected systematically with a microdebrider initially to debulk the tumor that was free-floating in the sinonasal cavity. The goal of the surgery is to identify the site(s) of attachment(s) so that the mucosa can be removed and the underlying bone drilled down to decrease the chance of tumor recurrence. Angled endoscopes, along with curved instruments, microdebriders and burrs were used for tumor resection within the maxillary sinus. A medial maxillectomy was performed to facilitate removal of the tumor origin (if it was attached to the medial maxillary sinus wall) or simply to allow increased access to all walls of the sinus cavity. For IPs originating from the anterior maxillary wall, a transseptal approach was used for increased angulation [16]. Frozen sections of resection margins were sent at the end of the operation to ensure complete removal of IP.

### Outcome measures

The primary outcome measures were IP attachment site(s) and recurrence. The number and location(s) of the IP attachment site(s) were determined based on direct visualization by the surgeon, and were extracted from the operative note. A secondary measure was the presence of malignant transformation including dysplasia.

### Statistical analysis

Descriptive statistics were used to summarize the frequency and percentage of categorical variables. Continuous variables are reported as mean and standard deviation (SD). Student’s t-test was performed to determine the differences between single and multiple sites of IP attachment for age, follow-up time, LOS, and disease recurrence. Fisher’s exact test was used to analyze differences in gender and previous recurrence. All statistical analyses were performed using Prism (v.7, GraphPad, USA), with significance set to α=0.05.

## Results

Twenty-eight maxillary IP patients (61% males) were identified and included for analysis, with a mean age of 54.9 (SD: 16.5) years. At a mean follow-up of 31.1 (SD: 22.6) months, no recurrences were identified. Ten patients (36%) were referred for IPs, which recurred after failing initial surgical treatment from another surgeon. Osteitis (36%) was commonly seen. Patient characteristics are shown in Table 1.

**Table 1:** Baseline Characteristics

Variables	Total Patient (*n* = 28)
Gender (male:female)	17:11
Age, years (mean, SD)	54.9 (16.5)
Prev. recurrence (n, %)	10 (36%)
Recurrence (n, %)	0 (0.0%)
Malignant transformation (n, %)	0 (0.0%)
Follow-up, months (mean, SD)	31.1 (22.6)
Length of stay, days (mean, SD)	0.68 (1.3)
Perioperative complications (n, %)	0 (0.0%)

Comparisons of baseline characteristics between single and multiple attachment sites IPs are shown in Table 2. There was no statistically significant difference in the rate of recurrence between single and multiple attachment IPs group prior to revision surgery. Additionally, no statistically significant difference was noted in follow-up time between single attachment IP patients, with a length of 37.6 (SD: 23.7) months, as compared to 25.4 (SD: 20.7) months in multifocal IP patients. LOS in hospital was short within both groups, and did not differ between groups.

**Table 2:** Comparison of Baseline Characteristics between Single and Multiple Attachment Sites

Variables	Single Attach. (*n* = 13)	Multiple Attach. (*n* = 15)	*P*-value
Gender (male:female)	7:6	10:5	0.700
Age, years (mean, SD)	55.8 (16.8)	54.2 (16.8)	0.807
Prev. recurrence (n, %)	3 (23%)	7 (47%)	0.254
Follow-up, months (mean, SD)	37.6 (23.7)	25.4 (20.7)	0.158
Length of stay, days (mean, SD)	0.31 (0.48)	1.0 (1.6)	0.145

Attachments of IP to the maxillary sinus walls were classified as either single (46%) or multiple (54%). IPs with single attachment predominately originated from the medial maxillary wall, while those with multiple pedicles involved primarily the medial, lateral, and anterior walls (Table 3). For maxillary IPs with multifocal sites of attachment, 67% of cases originated from 2 to 3 walls of the sinus cavity, but can also have multiple attachments to a single wall (Table 4).

**Table 3:** Wall of Maxillary Sinus Involved in Cases with Single and Multiple Attachment Sites

Maxillary Sinus Wall	Single Attach.	Multiple Attach.
Posterior	1	7
Inferior	0	7
Lateral	2	9
Anterior	1	9
Medial	8	10
Superior	1	3

**Table 4:** Number of Maxillary Sinus Walls Involved in Cases of Multifocal Attachment

Number of Walls Involved	Cases
1	2
2	5
3	5
4	1
5	0
6	2

All patients were managed with a pure endoscopic approach, with all requiring an endoscopic medial maxillectomy. No adjunctive external approaches were required and no perioperative complications were encountered.

## Discusssion

The complete resection of maxillary sinus IPs is crucial due to the propensity for recurrence and its malignancy risk. During surgery, the tumor must be followed down to its origin to facilitate a wide local excision of the pedicle site [7, 17, 18]. In our series, IPs were followed to their attachments, with diseased mucosa resected and bone drilling performed at the pedicle sites. The number of attachment sites can theoretically be associated with tumor recurrence, since most IP recurrences occur at the pedicle and multifocal IPs may be more difficult to manage surgically [7, 19]. Previous reports have demonstrated successful surgical results stemming from a pure endoscopic approach for maxillary IPs originating from the medial, superior, and posterior walls [4, 7, 20, 21]. However, endoscopic access to the anterior and inferior walls was noted to be more challenging, inadequate for reaching all of the tumor, which often necessitated adjunctive external approaches [7, 21–23].

Dean et al.*,* (2014) reported that using a transseptal surgical approach in combination with medial maxillectomy facilitated full visualization of anterolateral maxillary IPs with multiple attachment sites, which allowed for the complete resection of the tumor pedicle [16]. During their study, with a mean follow-up of 29 months, no recurrences were noted for both the single and multiple IP attachment site groups [16]. Compared to results from our study, this is a similar findings as we noted no disease recurrence between single and multifocal sites at 31.1 months, while using a similar transseptal approach for accessing the anterior maxillary wall. However, contrasting our findings of no perioperative complications, Dean et al. reported infraorbital paresthesia in 9% of patients, secondary to tumor invasion of the nerve [16].

In a study by Hong et al. (2015), evaluating surgical approaches used in resecting maxillary IPs, only 16.1% of patients underwent a pure endoscopic approach [7]. External approaches, including Canine fossa puncture via Caldwell-Luc approach or Caldwell-Luc operation, were added gradually as required if endoscopy was insufficient in resecting all parts of the IP. External approaches were mainly added for lateral, anterior, and inferior wall involvement [7]. Hong et al. showed that 48.4% of maxillary IPs originated from the anterior and/or inferior walls, comparable to 42.9% in our study [7]. Similar to our results, no recurrences were reported in patients who underwent a pure endoscopic approach at a mean follow-up of 64.2 months [ 7]. Of interest, the recurrence rate was found to be 9.7% in patients with IPs of multiple attachment sites, who underwent adjunctive external approaches [7]. In our experience, we found that an external approach is not necessary for access to all IP attachment sites. Instead, an endoscopic medial maxillectomy and use of angled scopes can facilitate visualization to the anterior and inferior maxillary walls.

The results of our study have several clinical implications. First, pure endoscopic management of sino-nasal disease decreases the need for prolonged LOS in hospital. A previous report by Sautter et al. (2007) demonstrated that patients who underwent endoscopic IP resection spent significantly fewer days in hospital as compared to patients who had open surgery [24]. Similarly, our study demonstrates low LOS of both our single and multifocal IPs. Other clinical implications of a pure endoscopic approach to maxillary sinus IPs have been well described in the literature and include avoidance of facial incision, minimizing scarring, decreased pain, swelling, and dysesthesia as compared to open surgery [20, 22]. Moreover, one of the largest single cohort studies on IP reported a reduction in recurrence with the implementation of endoscopic approaches [25]. These results were reiterated by a recent meta-analysis, which noted that endoscopic management of IPs reduced recurrence risk by 44% as compared to external approaches [26]. Additionally, endoscopic medial maxillectomy has been described as effective and reproducible for IP resection, with decreased operating time and morbidity as compared to open maxillectomy [27]. As we noted no perioperative complications within our series, our results may provide further evidence indicating the low morbidity stemming from the endoscopic approach.

This study has limitations. While we aimed to acquire long-term data on IP recurrence within our cohort, with a mean follow-up of 31.1 months, this still may not have adequately captured all cases of recurrence. It has been reported that up to 20% of recurrences may occur 5 years after resection for all IPs [28]. However, specific for maxillary IPs, recurrences have been noted to occur within a mean time of 20 months [7]. This study was also retrospective in nature, and has limitations inherent to such analysis. Our analysis focused on surgical outcomes by distinguishing single from multiple attachment sites IPs. With a larger sample size, analysis may be performed for surgical outcomes based on the exact number of attachments. Future studies should aim to assess longer-term outcomes.

## Conclusion

No differences in recurrence were noted between single and multifocal attachment maxillary IPs. The majority of IPs originating from the maxillary sinus frequently had multi-focal attachments. Despite surgical challenges of reaching all sinus walls, maxillary IPs may be managed effectively via a pure endoscopic approach.

## Abbreviations

IP: Inverted papilloma
LOS: Length of stay
SD: Standard deviation
